# Serine/threonine-protein kinase STK24 induces tumorigenesis by regulating the STAT3/VEGFA signaling pathway

**DOI:** 10.1016/j.jbc.2023.102961

**Published:** 2023-01-28

**Authors:** Senyan Lai, Dao Wang, Wei Sun, Xiaonian Cao

**Affiliations:** 1 Department of Gastrointestinal Surgery Center, Tongji Hospital, Tongji Medical College, Huazhong University of Science and Technology, Wuhan, China; 2 Department of Thoracic Surgery, Tongji Hospital, Tongji Medical College, Huazhong University of Science and Technology, Wuhan, China

**Keywords:** STK24, STAT3, angiogenesis, lung cancer, VEGFA, IHC, immunohistochemistry, NSCLC, nonsmall cell lung cancer, STK24, serine/threonine-protein kinase 24

## Abstract

Lung cancer is the most common cause of cancer-related death. Although anti-angiogenesis therapy has been effective in the treatment of nonsmall cell lung cancer (NSCLC), drug-resistance is a common challenge. Therefore, there is a need to develop new therapeutic strategies for NSCLC. Serine/threonine-protein kinase 24 (STK24), also known as MST3, belongs to the germinal center kinase III subfamily, and the biological function of STK24 in NSCLC tumorigenesis and tumor angiogenesis is still unclear. In this study, we demonstrated that STK24 was overexpressed in lung cancer tissues compared with normal lung tissues, and lung cancer patients with higher STK24 expression levels had shorter overall survival time. In addition, our *in vitro* assays using A549 and H226 cell lines revealed that the STK24 expression level of cancer cells was positively correlated with cancer cells proliferation, migration, invasion, and tumor angiogenesis ability; *in vivo* assays also demonstrated that silencing of STK24 dramatically inhibited tumor progress and tumor angiogenesis. To investigate a mechanism, we revealed that STK24 positively regulated the signal transducer and activator of transcription 3 (STAT3)/vascular endothelial growth factor A (VEGFA) signaling pathway by inhibiting polyubiquitin-proteasomal–mediated degradation of STAT3. Furthermore, we performed *in vivo* assays in BALB/c nude mice and *in vitro* assays to show that STK24-regulated tumor angiogenesis depends on STAT3. These findings deepened our understanding of tumor angiogenesis, and the STK24/STAT3/VEGFA signaling pathway might be a novel therapeutic target for NSCLC treatment.

Globally, lung cancer is the most common cause of cancer-related deaths, with almost 1.6 million deaths reported annually (1). Nonsmall cell lung cancer (NSCLC) accounts for 85 % of all the lung cancers. Further, it has been found that the most common histological subtypes are lung adenocarcinoma and lung squamous cell carcinoma accounting for approximately 25 to 30% as well as 40%, respectively (2). Surgery, radiotherapy, chemotherapy, targeted therapy, and immunotherapy are the main therapeutic strategies for treatment of patients with lung cancer (1, 3). Although anti-angiogenesis therapies, such as use of bevacizumab and ramucirumab, are effective for treatment of advanced stages of lung cancer and significantly improves prognosis of the patients, primary and secondary drug-resistance is a common problem with the strategy (4, 5, 6). To overcome the challenge, there is need for further studies toward understanding of tumor angiogenesis.

Tumor progression is often accompanied by tumor angiogenesis (7), which was first suggested by Folkman in 1971. It has been reported that anti-angiogenesis therapy greatly improves the outcomes of patients with cancer (8). Solid tumor vessels are immature, tortuous, and disorganized as well as excessively leaky. Excess leaking of the vessels results in high interstitial fluid pressure and a reduction in blood perfusion as well as oxygenation (4). During cancer progression, upregulation of multiple pro-angiogenic factors, such as vascular endothelial growth factor A (VEGFA), PDGF, and IGF-1, and downregulation of anti-angiogenic factors, such as ENS and PF-4, contributes to pathological angiogenesis (9, 10). Recent studies have shown that the redox state of cancer cells is a critical regulatory factor of tumor angiogenesis (11). The imbalance between pro-angiogenic and anti-angiogenic factors leads to development of immature vascular supply. Several classical signaling pathways, such as IL-6/signal transducer and activator of transcription (STAT3), HIF1/VEGFA, and PI3K/AKT, have been shown to regulate tumor angiogenesis (4, 8).

Serine/threonine-protein kinase 24 (STK24), also known as MST3, belongs to the subfamily germinal center kinase III, together with other members including STK4 (MST1), STK3 (MST2), andSTK26 (MST4) (12). STK24 is associated with cellular proliferation, differentiation, death, polarity, and exocytosis (12). For solid tumors, STK24 positively regulates ERK1/2 activation and cooperates with STK26 and YSK1 to promote migration and metastasis of the cancer cells (13, 14). The expression of STK24 is negatively correlated with the overall survival in patients with breast cancer (15). Furthermore, overexpression of STK24 is associated with chemoresistance to cisplatin, carboplatin, paclitaxel, etoposide, and erlotinib (16). Besides, STK24 can also regulate excitatory synaptic transmission in epileptic hippocampal neurons, inhibit cavernoma development, control kidney water reabsorption by regulating Aqp2 membrane targeting, and protect the obesity-associated metabolic disorders by disrupting the NLRP3 inflammasome (13, 14, 17, 18). However, the biological function of STK24 in NSCLC tumorigenesis and tumor angiogenesis is still unclear.

The present study demonstrated that STK24 acts as an oncogene in NSCLC tumorigenesis and positively regulates the proliferation, migration, and invasion potential of NSCLC cells. In addition, *in vivo* xenograft assays showed that loss of STK24 inhibited tumorigenesis. For mechanism, of action, the present study evidently revealed that overexpression of STK24 stabilizes and upregulates expression of STAT3 by decreasing the ubiquitination of STAT3. Moreover, it was evident that STK24-mediated regulation of tumor angiogenesis and proliferation was dependent on expression of STAT3. Therefore, results of the current study enhances the available understanding on tumor angiogenesis and shows that STK24/STAT3/VEGFA signaling pathway may be a novel therapeutic target for treatment of patients with NSCLC.

## Results

### STK24 functioned as an oncogene in NSCLC

**Figure 1:**
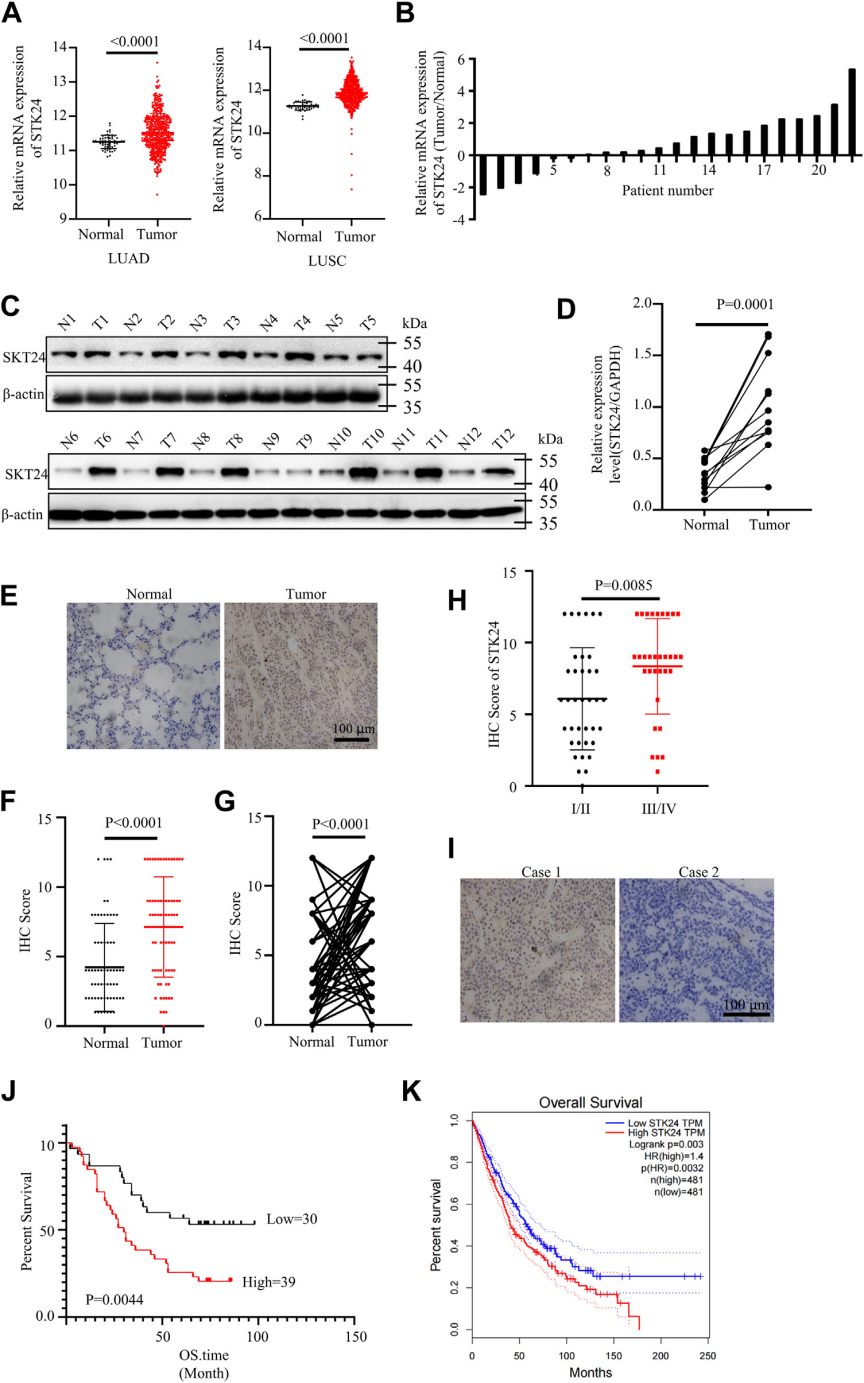
**STK24 acted as an oncogene in NSCLC.***A*, analysis of STK24 expression levels in normal lung tissues and NSCLC tissues using TCGA database (LUAD: normal = 59; tumor = 483; LUSC: normal = 51; tumor = 500); *Left p* < 0.0001, *Right p* < 0.0001, by Student’s *t* test. *B*, expression level analysis of STK24 in 22 pairs of NSCLC tissues and matched adjacent normal tissues using RT-PCR. *C*, immunoblots analysis of 12 pairs of NSCLC tissues and matched adjacent normal tissues. *D*, showing statistical analysis of *C* (n = 12); *p* = 0.0001, by Student’s *t* test. *E*, representative images of IHC assays for 69 pairs of NSCLC tissues and matched adjacent normal tissues; scale bar =100 μm. *F* and *G*, statistical analysis for IHC assay of NSCLC tissues and matched adjacent normal tissues in *E* (n = 69); *F* for *p* < 0.0001, by unpaired Student’s *t* test, *G* for *p* < 0.0001, by paired Student’s *t* test. *H*, statistical analysis for IHC assay of NSCLC tissues from patients grouped by clinical stage in *E* (n = 69); *p* = 0.0085. *I*, representative images of IHC assays for 69 NSCLC tissues; scale bar =100 μm. *J*, Kaplan-Meier curve of NSCLC patients grouped by IHC scores (high = 39 cases; low = 30 cases); *p* = 0.0044, by log-rank (Mantel-Cox) test. *K*, Kaplan-Meier curve of NSCLC patients grouped by STK24 expression levels using TCGA database (high = 481 cases; low = 481 cases); *p* = 0.0032, by Log-rank (Mantel-Cox) test. All immunoblots were conducted three time, and consistent results were found. IHC, immunohistochemistry; LUAD, lung adenocarcinoma; LUSC, lung squamous cell carcinoma; NSCLC, nonsmall cell lung cancer; STK24, serine/threonine-protein kinase 24; TCGA, The Cancer Genome Atlas.

The role of STK24 in tumorigenesis was investigated by first comparing the expression of STK24 between normal and NSCLC tissues using data obtained from The Cancer Genome Atlas (TCGA) database. Remarkably, it was found that STK24 was upregulated in both lung adenocarcinoma and lung squamous cell carcinoma (Fig. 1*A*). RT-PCR assays was then used to measure the mRNA expression levels of STK24 in 22 paired clinical NSCLC tissues and matched adjacent normal lung tissues.

**Supplemental Fig. S1:**
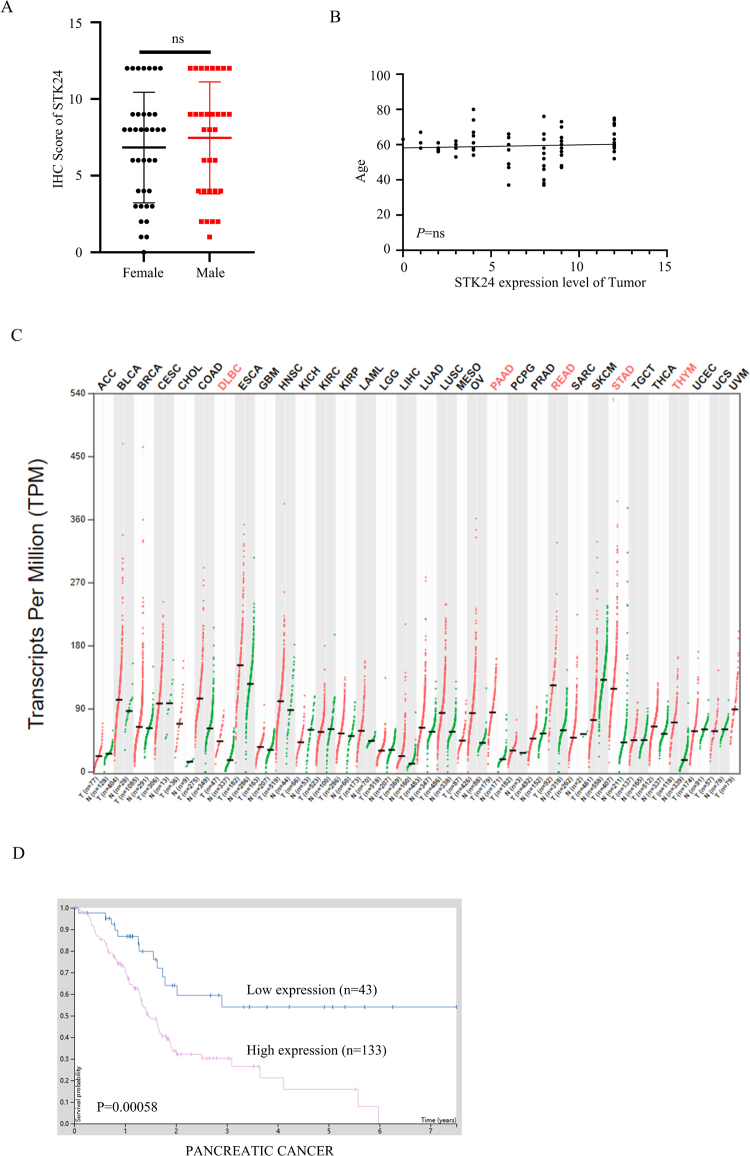
**STK24 was a prognostic risk factor for NSCLC patients.***A*, IHC analysis of NSCLC tissues of patients grouped by gender (n = 69), *p* = ns, by Student’s *t* test. *B*, the correlation of STK24 expression level of NSCLC with age of patients (n = 69) *p* = ns, by Pearson's r test. *C*, analysis of STK24 expression level in different types of cancer and normal tissues using GEPIA database. *D*, Kaplan–Meier curve of patients with pancreatic cancer grouped by STK24 expression level using TCGA database (low = 43; high = 133); *p* =0.00058 by Log-rank (Mantel–Cox) test. IHC, immunohistochemistry; NSCLC, nonsmall cell lung cancer; STK24, serine/threonine-protein kinase 24.

Results of the study showed that NSCLC tissues had higher STK24 expression level as compared with the matched adjacent normal lung tissues (Fig. 1*B*). The RT-PCR results were consistent with that of the Western blot and immunohistochemistry (IHC) (Fig. 1, *C*–*G*). Furthermore, patients with higher clinical stages had higher STK24 expression level, whereas the expression level of STK24 had no correlation with age or gender of the patients (Figs. 1*H* and S1, *A* and *B*). The STK24 expression levels were also higher in tissues of other cancers types as compared with the matched normal tissues (Fig. S1*C*).

The present study also analyzed the STK24 expression levels of 69 NSCLC tumor tissues using IHC assays. The patients were hence grouped according to the levels of STK24 expression, and survival analysis was carried out using Kaplan–Meier curves. Results of the analysis showed that patients with higher STK24 expression level was associated with poor overall survival time as compared with those with lower STK24 expression level (Fig. 1, *I* and *J*). Similar results were obtained after analysis of data for NSCLC and pancreatic cancer in the TCGA and Human Protein Atlas database (Figs. 1*K* and S1*D*). Overall, the findings of the current study showed that STK24 acts as an oncogene in NSCLC tumorigenesis.

### High STK24 expression promoted proliferation, migration, and invasion of NSCLC cells

**Figure 2:**
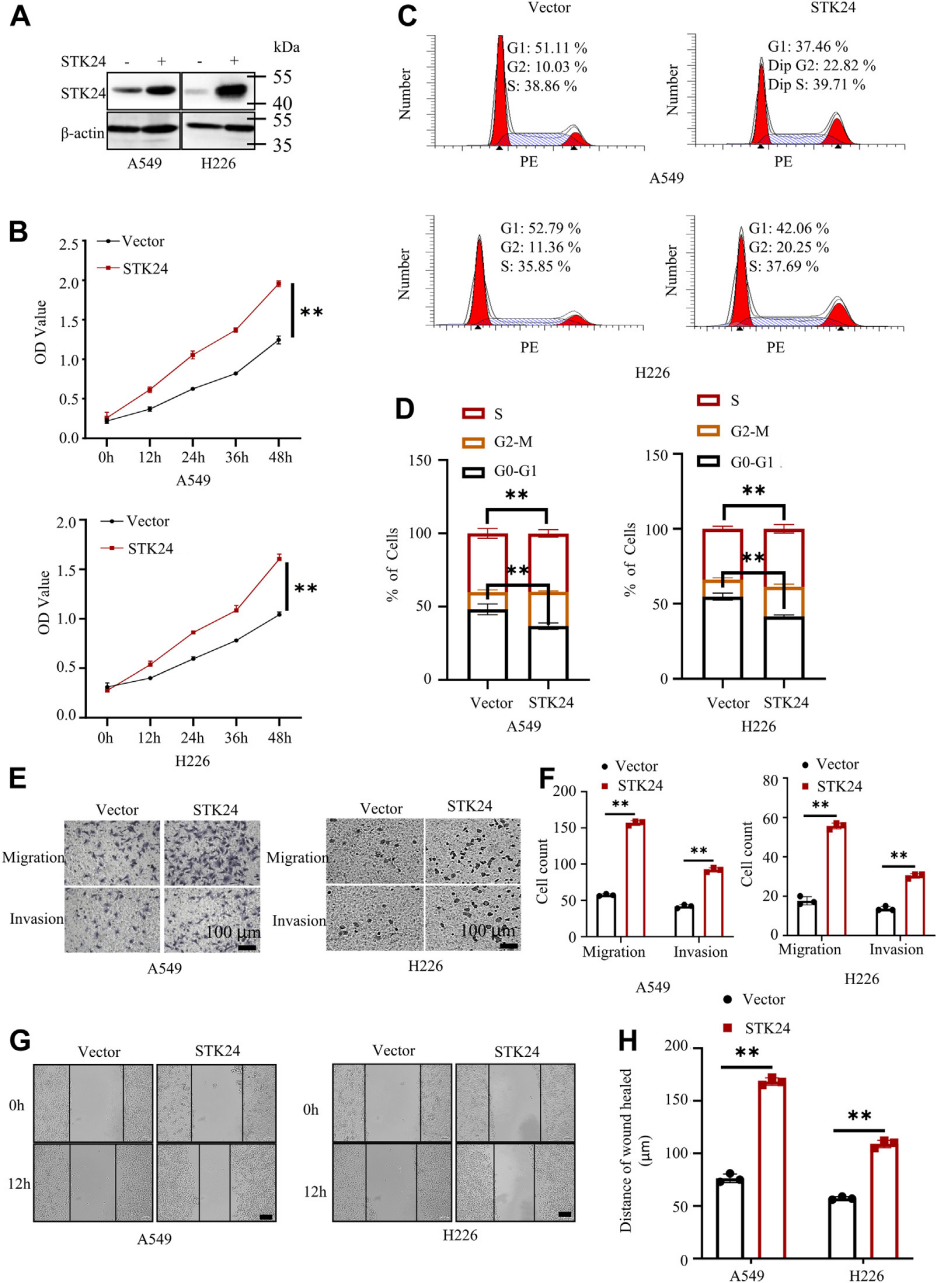
**STK24 overexpression promoted proliferation, migration, and invasion ability of NSCLC cells.***A*, immunoblot analysis of protein expression levels in NSCLC cells stably expressing vector or STK24. *B*, cell proliferation analysis of cells stably expressing vector or STK24 using CCK8 assays (n = 3); *p* < 0.0001, by Student’s *t* test. *C*, representative image of cell cycle analysis for cells stably expressing Vector or STK24. *D*, statistical analysis of cell cycle analysis in *C* (n = 3); *p* for *left* = 0.0095, by Student’s *t* test, *p* for *right* = 0.0009, by Student’s *t* test. *E*, representative images for transwell assays for NSCLC cells stably expressing vector or STK24; scale bar = 100 μm. *F*, statistical analysis of transwell assays in *E* (n = 3); *p* < 0.0001, by Student’s *t* test. *G*, representative images for wound healing assays for NSCLC cells stably expressing vector or STK24; scale bar =100 μm. *H*, statistical analysis of wound healing assays in *G* (n = 3); *p* < 0.0001, by Student’s *t* test. All immunoblots were conducted three times, and consistent results were found. NSCLC, nonsmall cell lung cancer; STK24, serine/threonine-protein kinase 24.

Two NSCLC cell lines: lung adenocarcinoma cancer cell line (A549) and lung squamous carcinoma cell line (H226) were selected for further experiments and assessment of the role of STK24 in NSCLC tumorigenesis. Lentiviruses was used to construct A546 and H226 cell lines with stable ectopic expression of vector or STK24, and Western blot analysis was performed to confirm the stable expression (Fig. 2*A*). The CCK8 assays were carried out in the present study to assess the proliferation potential of vector or STK24 NSCLC cell lines. It was found that the stable expression of STK24 significantly enhanced proliferative potential of NSCLC cells (Fig. 2*B*). Results of cell cycle analysis revealed that cells expressing STK24 had a higher S and G2-M phase and a lower G0-G1 phase as compared with cells transfected with the vector (Fig. 2, *C* and *D*). The findings showed that overexpression of STK24 enhances the proliferation ability of NSCLC cells.

**Supplemental Fig. S2:**
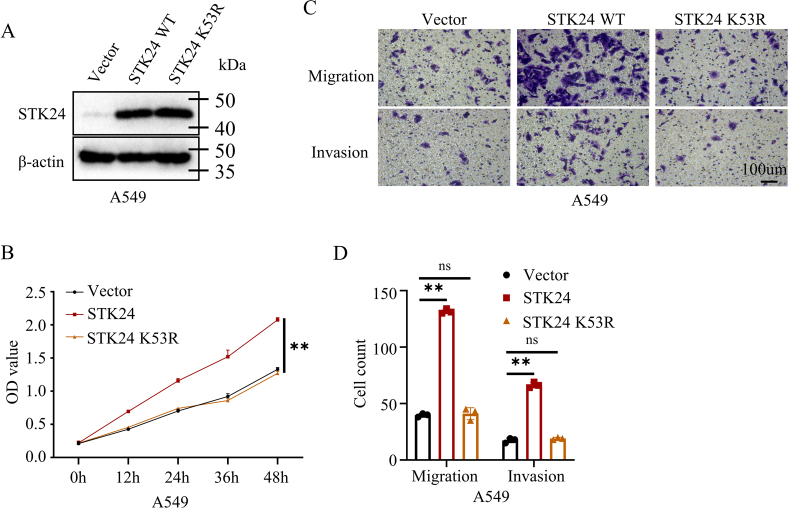
**The kinase activity of STK24 was required for its effects on invasion, migration, and proliferation.***A*, immunoblot analysis of indicated protein expression levels in NSCLC cells ectopic expressing vector, STK24 WT, or STK K53R. *B*, cell proliferation analysis of cells ectopic expressing vector, STK24 WT, or STK K53R (n = 3); *p* < 0.0001, by Student’s *t* test. *C*, representative images of transwell assays for NSCLC cells ectopic expressing vector, STK24 WT, or STK K53R; scale bar = 100 μm. *D*, statistical analysis of transwell assays in Fig. S2C (n = 3); *p* < 0.0001 (vector *versus* STK24 WT) or ns (vector *versus* STK24 K53R), p = ns, (STK24 WT *versus* STK24 K53R), by Student’s *t* test. All immunoblots were conducted three times, and consistent results were found. NSCLC, nonsmall cell lung cancer; STK24, serine/threonine-protein kinase 24.

Tumor progression is associated with increased potential of cancer cells metastasis. Therefore, transwell assays were performed to assess the metastasis potential of cells with stable expression of STK24. It was found that STK24 overexpression promotes invasion and migration of cancer cells (Fig. 2, *E* and *F*), which was consistent with the results obtained in the wound healing assays (Fig. 2, *F* and *G*). Furthermore, we constructed a kinase activity-deficient STK24 mutant (STK24 K53R) (19, 20) and proved that the kinase activity of STK24 was required for its effects on invasion, migration, and proliferation of cancer cells (Fig. S2, *A*–*D*) Overall, the results of the current study demonstrated that cancer cells with higher STK24 expression have a higher proliferation, migration, and invasion potential.

### Loss of STK24 expression inhibits proliferation, migration, and invasion of cancer cells

**Figure 3:**
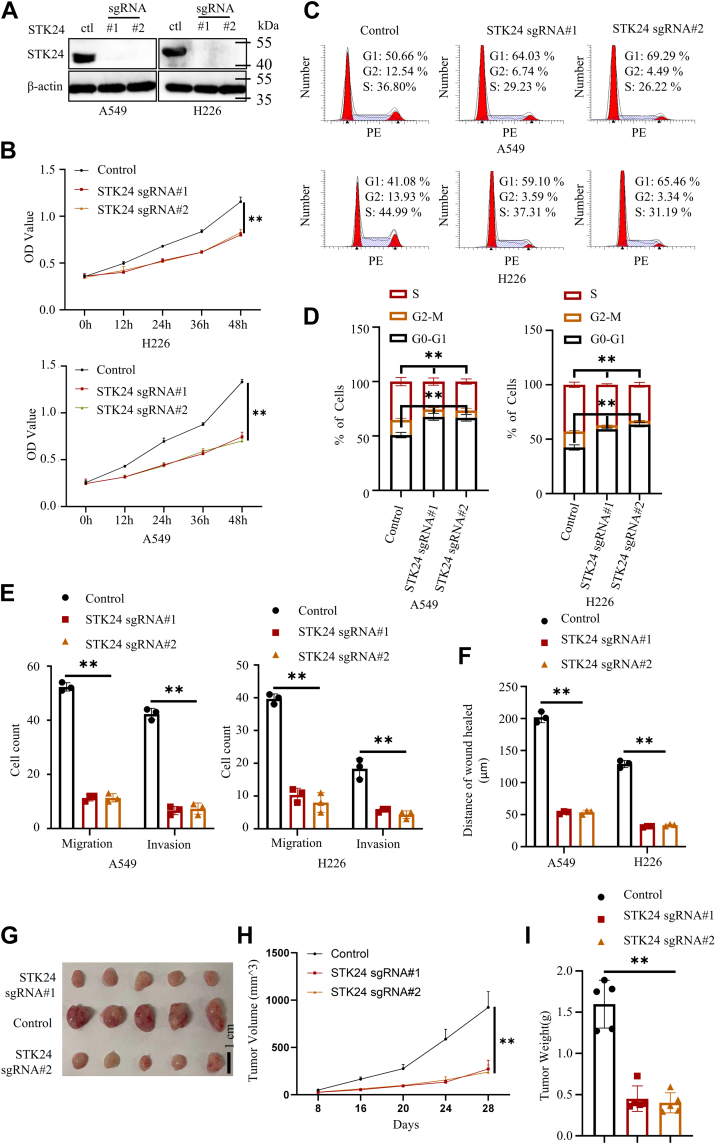
**Loss of STK24 inhibited proliferation, migration, and invasion ability of NSCLC cells.***A*, immunoblot analysis of protein expression levels in NSCLC cells stably expressing control, sgSTK24#1, or sgSTK24#2. *B*, cell proliferation analysis of cells stably expressing control, sgSTK24#1, or sgSTK24#2 using CCK8 assays (n = 3); *p* < 0.0001 (control *versus* sgRNA#1 or sgRNA#2), by Student’s *t* test. *C*, representative image of cell cycle analysis for cells stably expressing control, sgSTK24#1, or sgSTK24#2. *D*, statistical analysis of cell cycle analysis in *C* (n = 3); *p* for *Left* = 0.0019 (control *versus* sgRNA#1), 0.0022 (control *versus* sgRNA#2), by Student’s *t* test, *p* for *right* = 0.0004 (control *versus* sgRNA#1), 0.0003 (control *versus* sgRNA#2), by Student’s *t* test. *E*, statistical analysis of transwell assays (shown in Fig. S3, *A* and *B*) for NSCLC cells stably expressing control, sgSTK24#1, or sgSTK24#2 (n = 3); *p* < 0.0001 (control *versus* sgRNA#1 or sgRNA#2), by Student’s *t* test. *F*, statistical analysis of wound healing assays (shown in Fig. S3, *C* and *D*) for NSCLC cells stably expressing control, sgSTK24#1, or sgSTK24#2; *p* < 0.0001 (control *versus* sgRNA#1 or sgRNA#2), by Student’s *t* test. *G*, mice xenograft experiments were conducted using NSCLC cells stably expressing control, sgSTK24#1, or sgSTK24#2, and representative tumor image shown. *H*, tumor growth curve shown in *G* (n = 5); *p* < 0.0001 (control *versus* sgRNA#1 or sgRNA#2), by Student’s *t* test. *I*, statistical analysis of tumor weight in *G* (n = 5); *p* < 0.0001 (control *versus* sgRNA#1 or sgRNA#2), by Student’s *t* test. All immunoblots were conducted three times, and consistent results were found. NSCLC, nonsmall cell lung cancer; STK24, serine/threonine-protein kinase 24.

**Supplemental Fig. S3:**
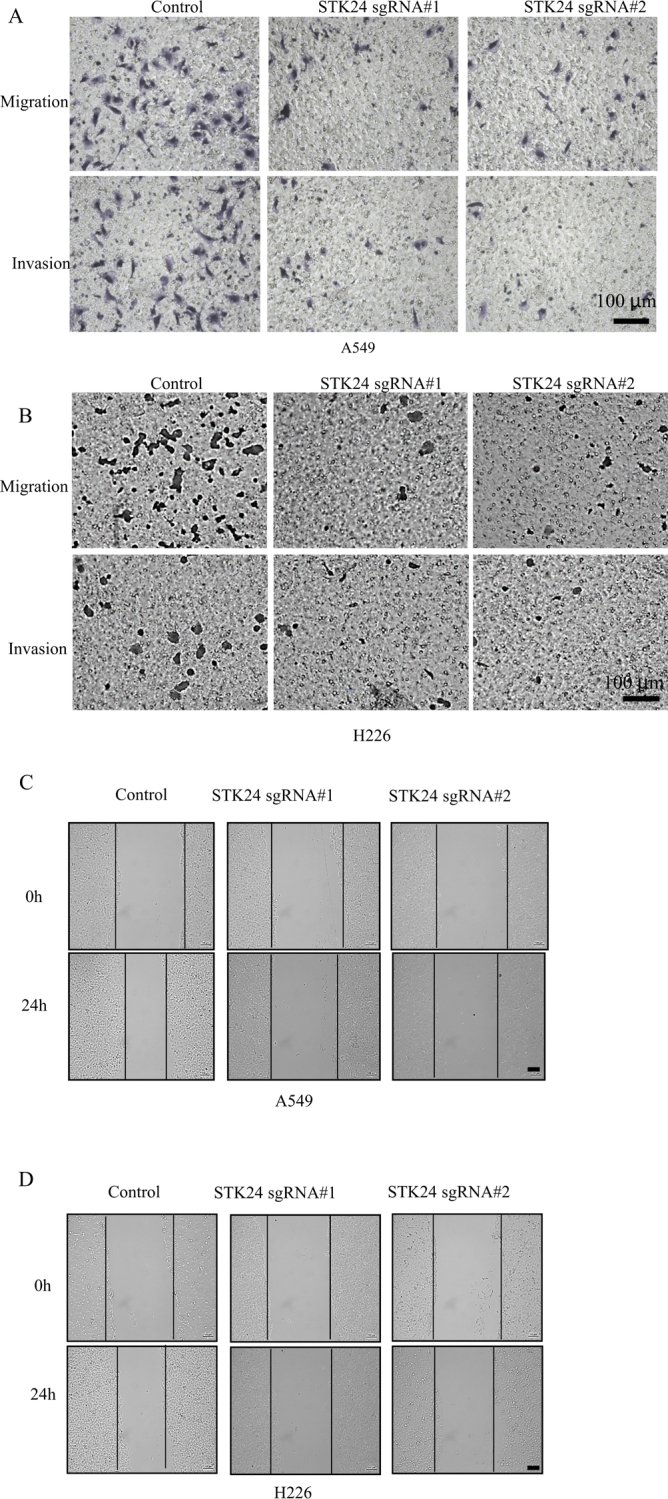
**Loss of STK24 inhibited NSCLC cell migration and invasion ability.***A* and *B*, representative images of transwell assays for NSCLC cells stably expressing control, sgSTK24#1, or sgSTK24#2; scale bar =100 μm. *C* and *D*, representative images of wound healing assays for indicated NSCLC cells stably expressing control, sgSTK24#1, or sgSTK24#2; scale bar =100 μm. NSCLC, nonsmall cell lung cancer; STK24, serine/threonine-protein kinase 24.

**Supplemental Fig. S4:**
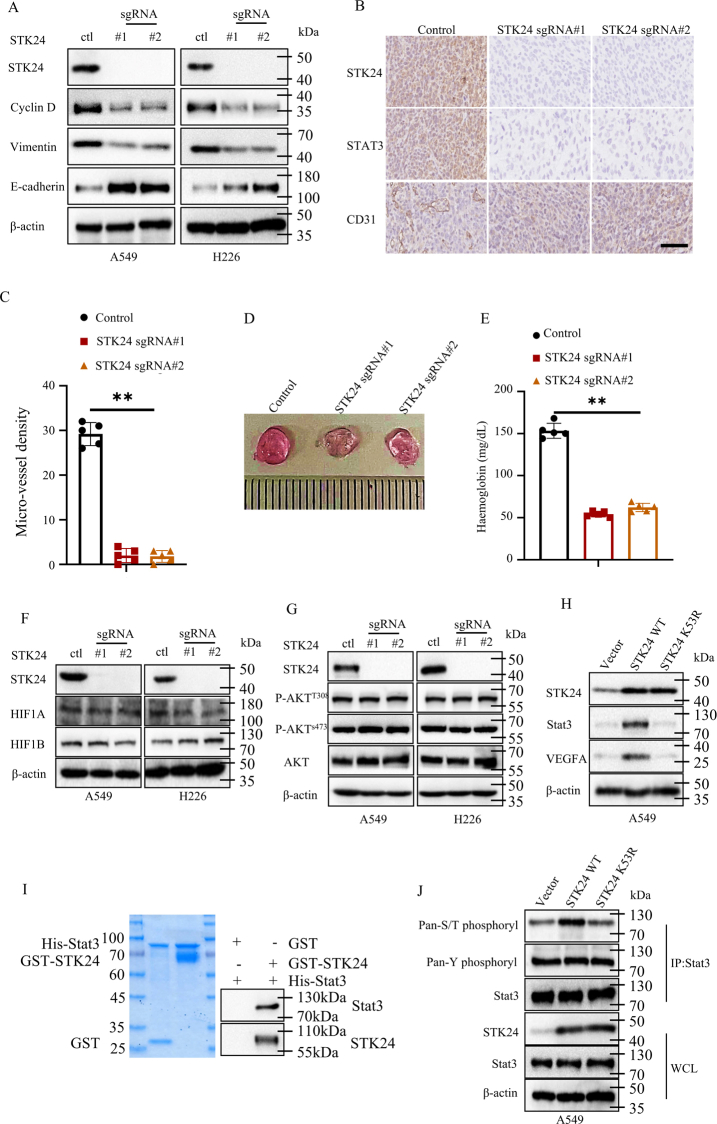
**STK24 regulated tumor angiogenesis.***A*, immunoblot analysis of indicated protein expression levels in NSCLC cells stably expressing control, sgSTK24#1, or sgSTK24#2. *B*, representative images for IHC analysis of tumors induced by NSCLC cells stably expressing control, sgSTK24#1, or sgSTK24#2; scale bar = 100 μm. *C*, statistical analysis of micro-vessel density in identified tumors (n = 5); *p* < 0.0001 (control *versus* sgRNA#1 or sgRNA#2), by Student’s *t* test. *D*, representative images for Matrigel plug assays. *E*, statistical analysis of hemoglobin measurement of indicated Matrigel in *D* (n = 5); *p* < 0.0001 (control *versus* sgRNA#1 or sgRNA#2), by Student’s *t* test. *F*–*H*, immunoblot analysis of indicated protein expression levels in pretreated NSCLC cells. *I*, the direct binding of GST-STK24 and His-STAT3 was investigated by GST-pull down assays. *J*, immunoprecipitation and immunoblot analysis of indicated protein expression level of NSCLC cells ectopic expressing vector, STK24 WT, or STK K53R. All immunoblots were conducted three time, and consistent results were found. NSCLC, nonsmall cell lung cancer; STK24, serine/threonine-protein kinase 24.

Expression of STK24 in NSCLC cell lines was knocked out using Crisp-Cas9 system to verify the findings obtained in the present study, and Western blot was used to confirm successful deletion of the gene (Fig. 3*A*). The CCK8 and cell cycle analysis were conducted in the current study to investigate the proliferation potential of the cells. Results showed that silencing of STK24 inhibited the proliferation of NSCLC cells. Further, it was evident that sgSTK24 NSCLC cells had a higher G0-G1 phase and a lower S and G2-M phase (Fig. 3, *B*–*D*). In addition, the findings of the transwell and wound healing assays showed that loss of STK24 significantly inhibited the migration as well as invasion potential of NSCLC cells (Figs. 3, *E* and *F* and S3, *A*–*D*). Moreover, it was revealed that loss of STK24 inhibited expression of cyclin D and vimentin and promoted expression of E-cadherin in the cancer cells (Fig. S4*A*).

In the mouse xenograft transplantation assay, A549 cells expressing control, sgSTK24#1, or sgSTK24#2 were subcutaneously injected into 4-week-old male nude BALB/c mice. Tumor volume was measured every after 4 days and after 28 days. All the mice were sacrificed, and their tumors were separated and weighed. Results showed that A549 cells without STK24 had slower growth rate as compared with control A549 cells (Fig. 3, *G*–*I*). Overall, the findings of the present study confirmed that STK24 was an oncogene in tumorigenesis.

### STK24 promoted tumor angiogenesis

The formation of tumor vessels enables supply of nutrients for growth of tumor, but the hypoxic environment in tumors leads to the formation of immature vessels (8). The formation of immature vessels in the tumors is called tumor angiogenesis. VEGFA is a key factor in the formation of the vessels (21). The formation of immature vessels has been shown to significantly promote tumor progression (4). Further, the anti-VEGF-VEGFRs axis has also been shown to be an efficient strategy for tumor treatment (22, 23).

**Figure 4:**
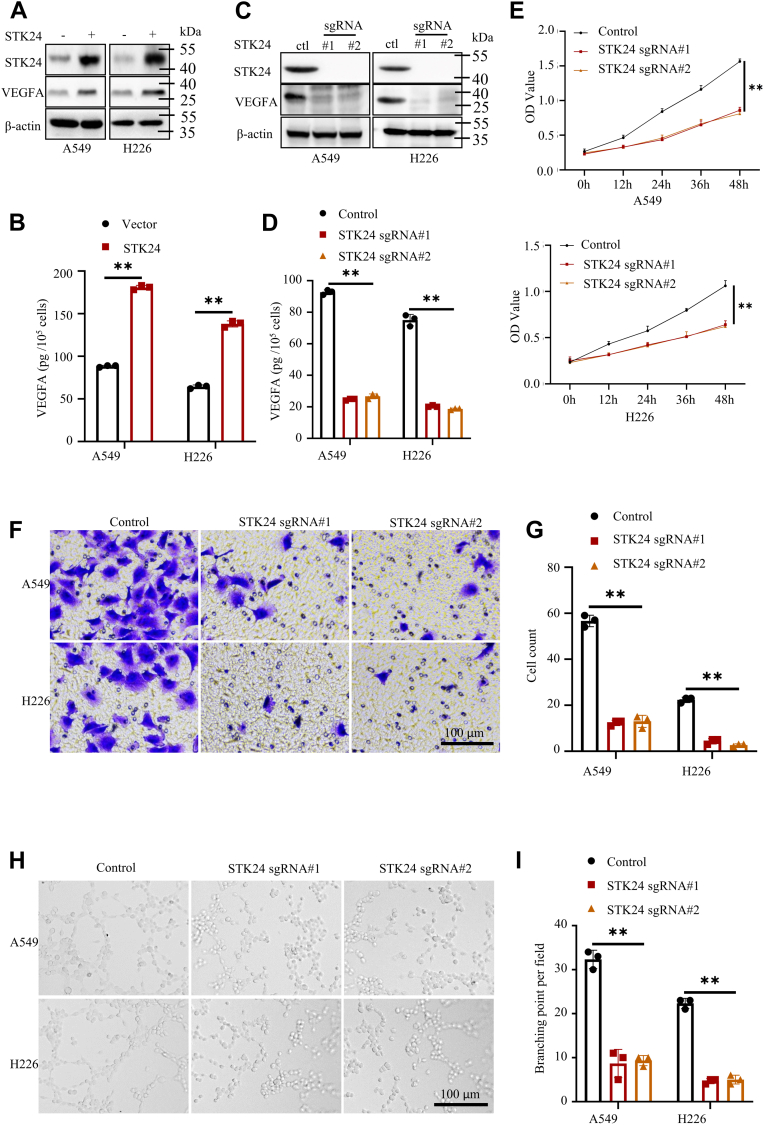
**STK24 regulated tumor angiogenesis.***A* and *C*, immunoblot analysis of protein expression levels in indicated NSCLC cells. *B* and *D*, statistical analysis of the expression levels of VEGFA using ELISA in indicated NSLCL cells (n = 3); *p* < 0.0001, by Student’s *t* test. *E*, cell proliferation analysis of HUVECs cultured with different CMs using CCK8 assays (n = 3); *p* < 0.0001 (control *versus* sgRNA#1 or sgRNA#2), by Student’s *t* test. *F*, representative images of trans-well assays for HUVECs cultured with different mediums; scale bar =100 μm. *G*, statistical analysis for trans-well assays in *F* (n = 3); *p* < 0.0001 (control *versus* sgRNA#1 or sgRNA#2), by Student’s *t* test. *H*, representative images of tube formation assays for HUVECs cultured with different mediums; scale bar =100 μm. *I*, statistical analysis for tube formation assays in *H* (n = 3); *p* < 0.0001 (control *versus* sgRNA#1 or sgRNA#2), by Student’s *t* test. All immunoblots were conducted three times, and consistent results were found. CM, conditional media; NSCLC, nonsmall cell lung cancer; STK24, serine/threonine-protein kinase 24.

The present study investigated the role of STK24 in tumor angiogenesis by assessing the density of microvessel in subcutaneous tumors when induced by A549 cells expressing control, sgSTK24#1, or sgSTK24#2. Notably, it was found that the sgSTK24 tumors had less microvessels as compared with control tumors (Fig. S4 *B*, and *C*). This implies that STK24 regulates tumor angiogenesis. Moreover, Matrigel plug assays were also conducted, and hemoglobin measurement indicated a decreased blood perfusion with loss of STK24 (Fig. S4 *D*, and *E*). The current study also investigated if STK24 regulates the secretion of VEGFA by cancer cells because VEGFA is an essential factor of tumor angiogenesis. Results of Western blot and enzyme-linked immunosorbent assay (ELISA) analysis revealed that overexpression of STK24 promotes secretion of VEGFA by cancer cells, whereas the loss of STK24 decreased the secretion of VEGFA by the cells (Fig. 4, *A*–*D*). Conditional media (CM) were then collected from control, sgSTK24#1, or sgSTK24#2 cancer cells and used them to culture human umbilical vein endothelial cells (HUVECs).

Results of CCK8 assays showed that HUVECs cultured with CMs from sgSTK24 cells had lower proliferation potential as compared with HUVECs cultured with CMs from control cells (Fig. 4*E*). Furthermore, results of transwell and tube formation assays revealed that HUVECs cultured with CMs from sgSTK24 cells had lower migration and tube formation ability as compared with those cultured with CMs from control cells (Fig. 4, *F*–*H*). Overall, the findings of the current study showed that STK24 positively regulates tumor angiogenesis.

### STK24 regulated STAT3/VEGFA signaling pathway

**Figure 5:**
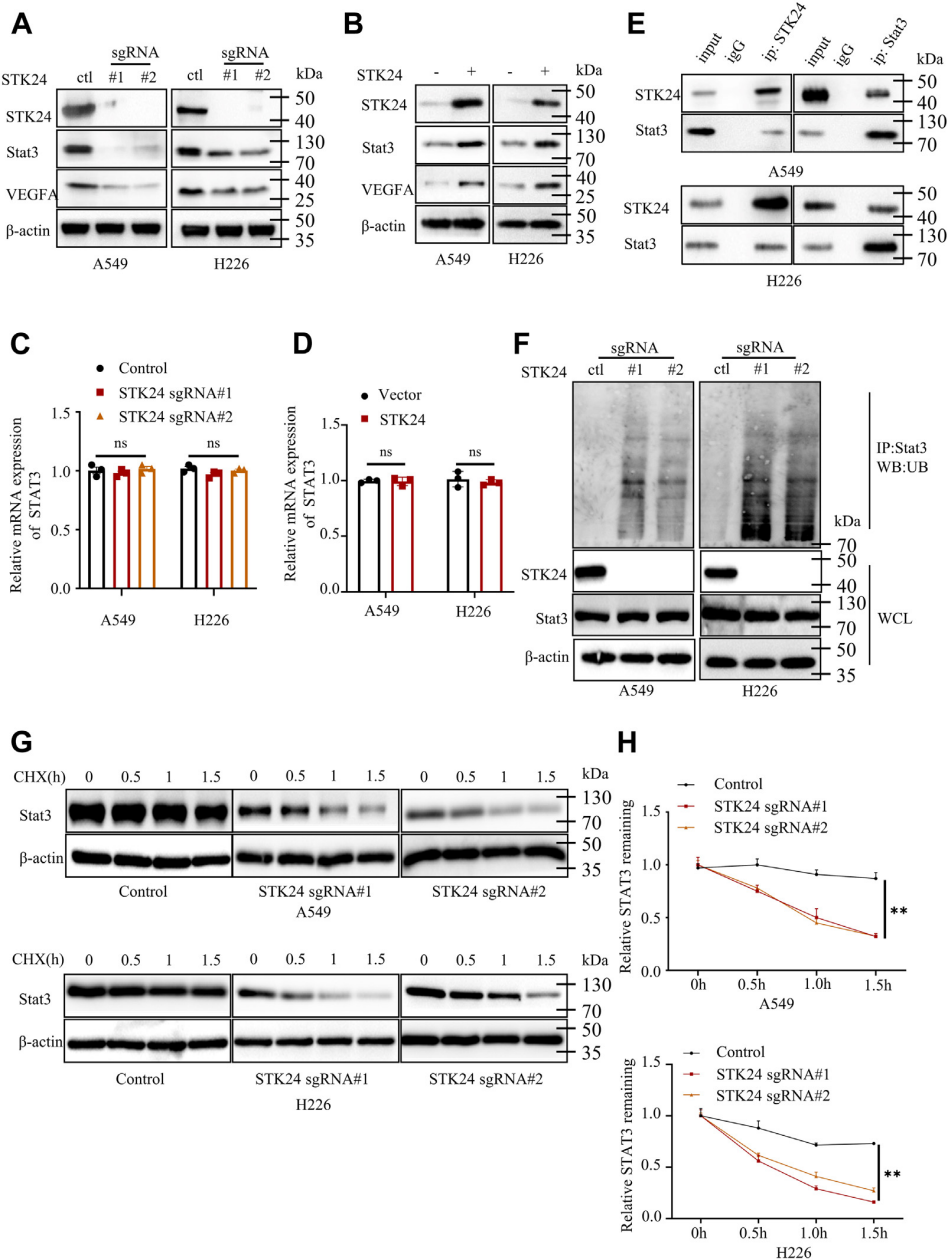
**STK24 regulated STAT3/VEGFA signaling pathway.***A* and *B*, immunoblot analysis of protein expression levels in the indicated NSCLC cells. *C* and *D*, STAT3 mRNA expression levels using RT-PCR assays in the indicated NSCLC cells (n = 3), *p* = ns (control *versus* sgRNA#1 or sgRNA#2), by Student’s *t* test. *E*, investigating the binding of STAT3 and STK24 using immunoprecipitation and immunoblot assays in indicated NSCLC cells. *F*, denaturing immunoprecipitation and immunoblot analysis of NSCLC cells transfected with HA-tagged ubiquitin plasmid. Cells were treated with MG132 before transfection to inhibit endogenous protein degradation. *G*, immunoblot analysis of NSCLC cells stably expressing control, sgSTK24#1, or sgSTK24#2, and all cells were treated with CHX to inhibit endogenous protein generation at indicated time points. *H*, statistical analysis for *G* (n = 3); *p* < 0.0001 (control *versus* sgRNA#1 or sgRNA#2), by Student’s *t* test. All immunoblots were conducted three time, and consistent results were found. CHX, cycloheximide; NSCLC, nonsmall cell lung cancer; STK24, serine/threonine-protein kinase 24.

Three signaling pathways (STAT3/VEGFA, HIF1/VEGFA, and AKT) have been reported to regulate tumor angiogenesis (24). To unravel the detailed mechanism of STK24-mediated tumor angiogenesis, the present study investigated whether STK24 could regulate the three signaling pathways. It was notable that loss of STK24 only decreased STAT3 expression but had no influence on HIF1A, HIF1B, AKT, and AKT phosphorylation (Figs. 5*A*, 4*F*, and S4*G*). The results were also confirmed by the analysis findings of the IHC assays (Fig. S4*B*).

Consistently, it was noted that the gain of STK24 increased expression levels of STAT3 in A549 and H226 cancer cells (Fig. 5*B*). Besides, STK24 kinase activity was required for STK24-mediated STAT3 and VEGFA expression upregulation (Fig. S4*H*). STAT3 protein expression levels are regulated at the transcriptional and posttranscriptional levels. Therefore, RT-PCR was first carried out to detect the mRNA expression levels of STAT3 in indicated cancer cells. Remarkably, it was found that the gain or loss of STK24 had no effect on the mRNA expression levels of STAT3 (Fig. 5, *C* and *D*). Results obtained from co-IP analysis revealed that there was a physical interaction between STK24 and STAT3 (Fig. 5*E*). Moreover, GST pull-down analysis proved that there was a direct physical interaction between STK24 and STAT3 (Fig. S4*I*). Upon these findings, we assumed that STK24 regulates STAT3 stabilization. Since ubiquitylation-dependent degradation is the main mode of posttranscriptional regulation of proteins, the ubiquitylation levels of STAT3 in control, sgSTK24#1, and sgSTK24#2 cancer cells were also investigated, and denaturing immunoprecipitation assays were performed under denaturing conditions to eliminate interacting proteins. MG132 was used to inhibit endogenous STAT3 degradation, and then, all cells were transfected with ha-ubiquitin plasmid to increase the ubiquitylation level of all endogenous proteins. Results of the immunoblot analysis showed that loss of STK24 significantly increased the ubiquitylation level of endogenous STAT3 (Fig. 5*F*). When cycloheximide was used to inhibit the generation of endogenous protein, it was noted that the degradation rate of STAT3 in sgSTK24 cells exceeded that in the control cells (Fig. 5, *G* and *H*). Since STK24 belonged to serine/threonine-protein kinase family, we further detect whether STK24 could regulate STAT3 phosphorylation level. MG132 was used to inhibit endogenous STAT3 degradation. Results showed that STK24 could regulate STAT3 serine/threonine phosphorylation level, but not tyrosinase phosphorylation level. In conclusion, the results of the present study demonstrated that loss of STK24 destabilizes STAT3 and further inhibits the STAT3/VEGFA signaling pathway.

### STK24-mediated tumor angiogenesis relied on STAT3/VEGFA signaling pathway *in vitro*

**Figure 6:**
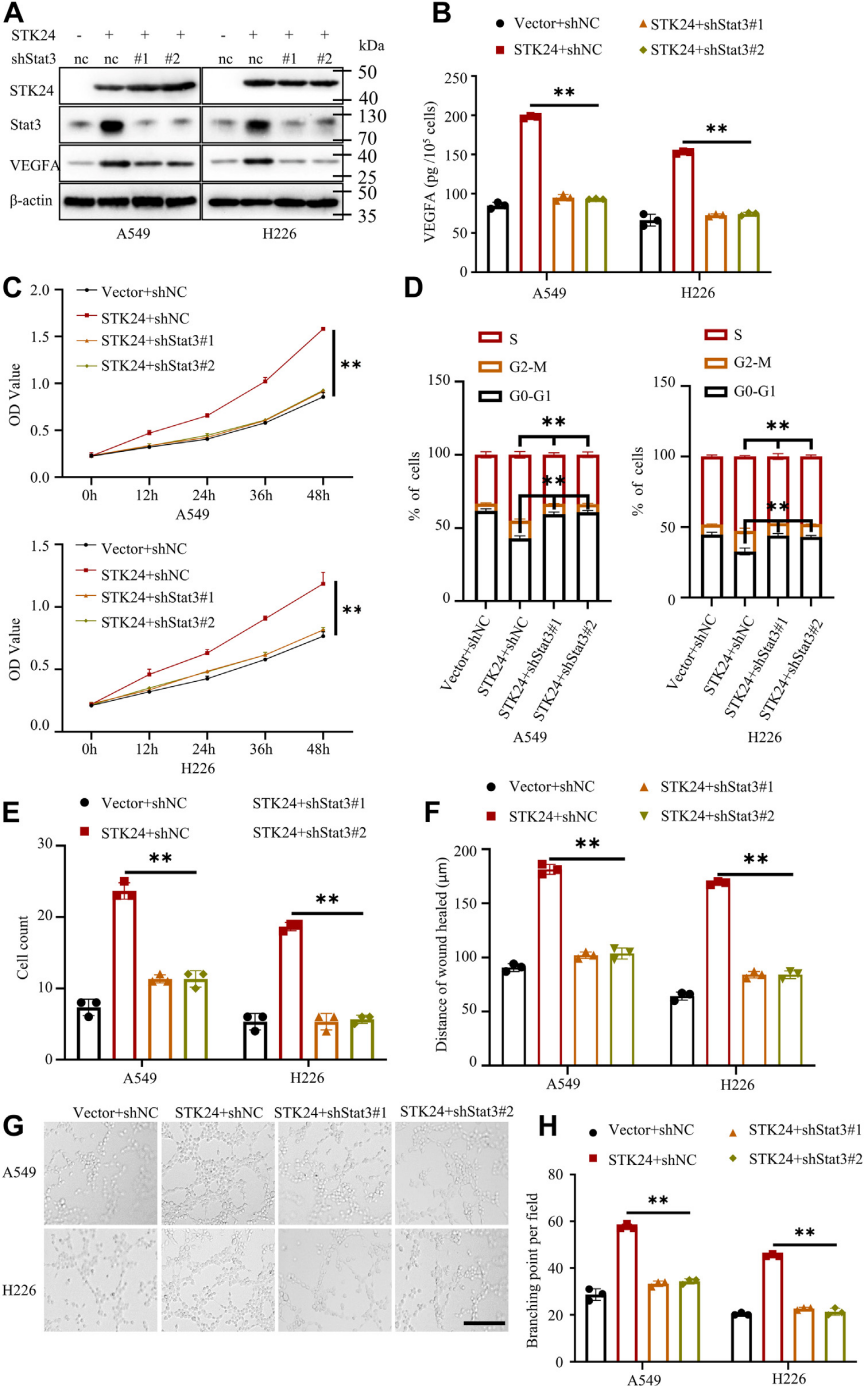
**STK24-mediated regulation of tumor angiogenesis was dependent on STAT3 *in vitro*.***A*, immunoblot analysis of indicated protein expression levels in NSCLC cells stably expressing vector+shnc, STK24+shnc, STK24+shSTAT3#1, or STK24+shSTAT3#2. *B*, statistical analysis of the VEGFA expression levels using ELISA (n = 3); *p* < 0.0001 (STK24+shnc *versus* STK24+shSTAT3#1 or STK24+shSTAT3#2), by Student’s *t* test. *C*, cell proliferation analysis of HUVECs cultured with different CMs using CCK8 assays (n = 3); *p* < 0.0001 (STK24+shnc *versus* STK24+shSTAT3#1 or STK24+shSTAT3#2), by Student’s *t* test. *D*, statistical analysis of cell cycle analysis in Fig. S5*A* (n = 3); *p* for *Left* = 0.0002 (STK24+shnc *versus* STK24+shSTAT3#1), 0.0001 (STK24+shnc *versus* STK24+shSTAT3#2), by Student’s *t* test, *p* for *right* = 0.0024 (STK24+shnc *versus* STK24+shSTAT3#1), 0.0025 (STK24+shnc *versus* STK24+shSTAT3#2), by Student’s *t* test. *E*, statistical analysis of trans-well assays in Fig. S5*B* (n = 3); *p* < 0.0001 (STK24+shnc *versus* STK24+shSTAT3#1 or STK24+shSTAT3#2), by Student’s *t* test. *F*, statistical analysis of wound healing assays in Fig. S5*C* (n = 3); *p* < 0.0001 (STK24+shnc *versus* STK24+shSTAT3#1 or STK24+shSTAT3#2), by Student’s *t* test. *G*, representative images for tube formation assays for HUVECs cultured with different conditional mediums; scale bar = 100 μm. *H*, statistical analysis of tube formation assays in *G* (n = 3); *p* < 0.0001 (STK24+shnc *versus* STK24+shSTAT3#1 or STK24+shSTAT3#2), by Student’s *t* test. All immunoblots were conducted three times, and consistent results were found. CM, conditional media; NSCLC, nonsmall cell lung cancer; STK24, serine/threonine-protein kinase 24.

**Supplemental Fig. S5:**
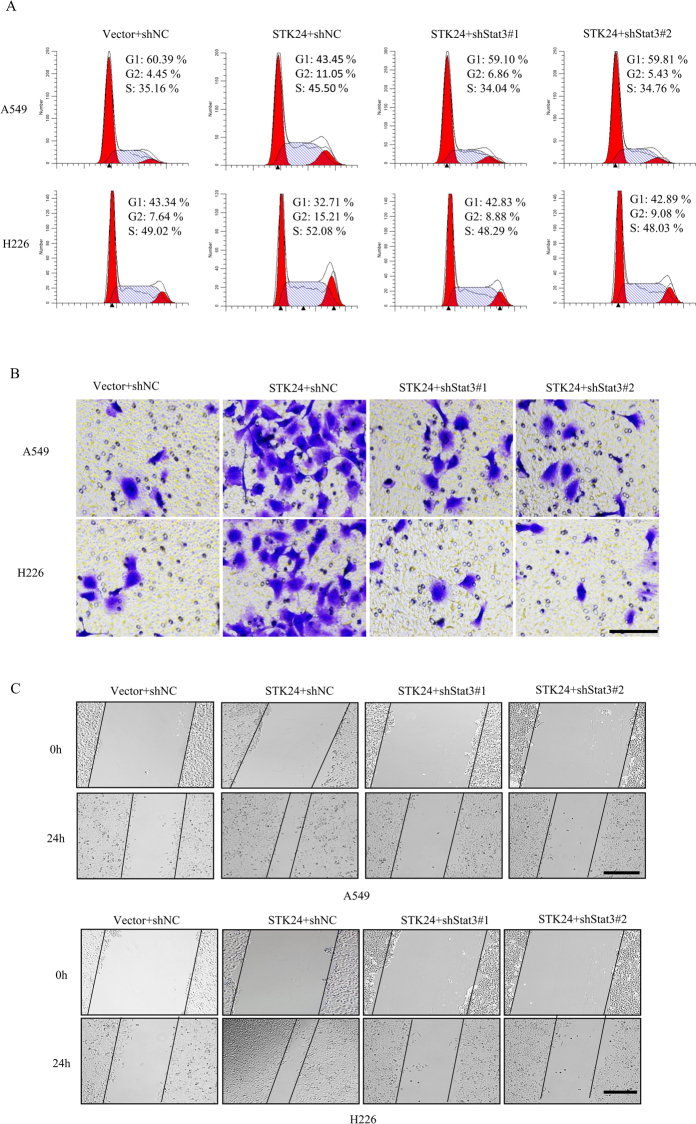
**STK24-mediated tumor angiogenesis relied on STAT3.***A*, representative image of cell cycle analysis for indicated cells. *B*, representative images of transwell assays for indicated NSCLC cells; scale bar =100 μm. *C*, representative images of wound healing assays for indicated NSCLC cells; scale bar = 100 μm. NSCLC, nonsmall cell lung cancer; STK24, serine/threonine-protein kinase 24.

To investigate the correlation between STK24 expression and STAT3/VEGFA signaling pathway during tumor angiogenesis, the present study constructed NSCLC cell lines stably expressing vector+shnc, STK24+shnc, STK24+shSTAT3#1, or STK24+shSTAT3#2 and used immunoblot analysis to confirm the stable expression (Fig. 6*A*). Results of the Western blot and ELISA assays showed that STK24-mediated VEGFA upregulation was reversed by silencing of STAT3 (Fig. 6, *A* and *B*). The CMs that were obtained from cancer cells stably expressing vector+shnc, STK24+shnc, STK24+shSTAT3#1, or STK24+shSTAT3#2 were used to maintain HUVECs. Results of cell cycle analysis, CCK8, transwell, wound healing, and tube formation assays showed that HUVECs cultured with CMs from STK24+shnc cells had higher proliferation, migration, and tube formation potential as compared with HUVECs cultured with CMs from the vector+shnc cells. However, this effect was abrogated by silencing the expression of STAT3 (Figs. 6, *C*–*H* and S5). The results of the current study revealed that STK24-mediated tumor angiogenesis was dependent on STAT3/VEGFA signaling pathway.

### Silencing of STAT3 inhibited STK24-induced tumor angiogenesis *in vivo*

**Figure 7:**
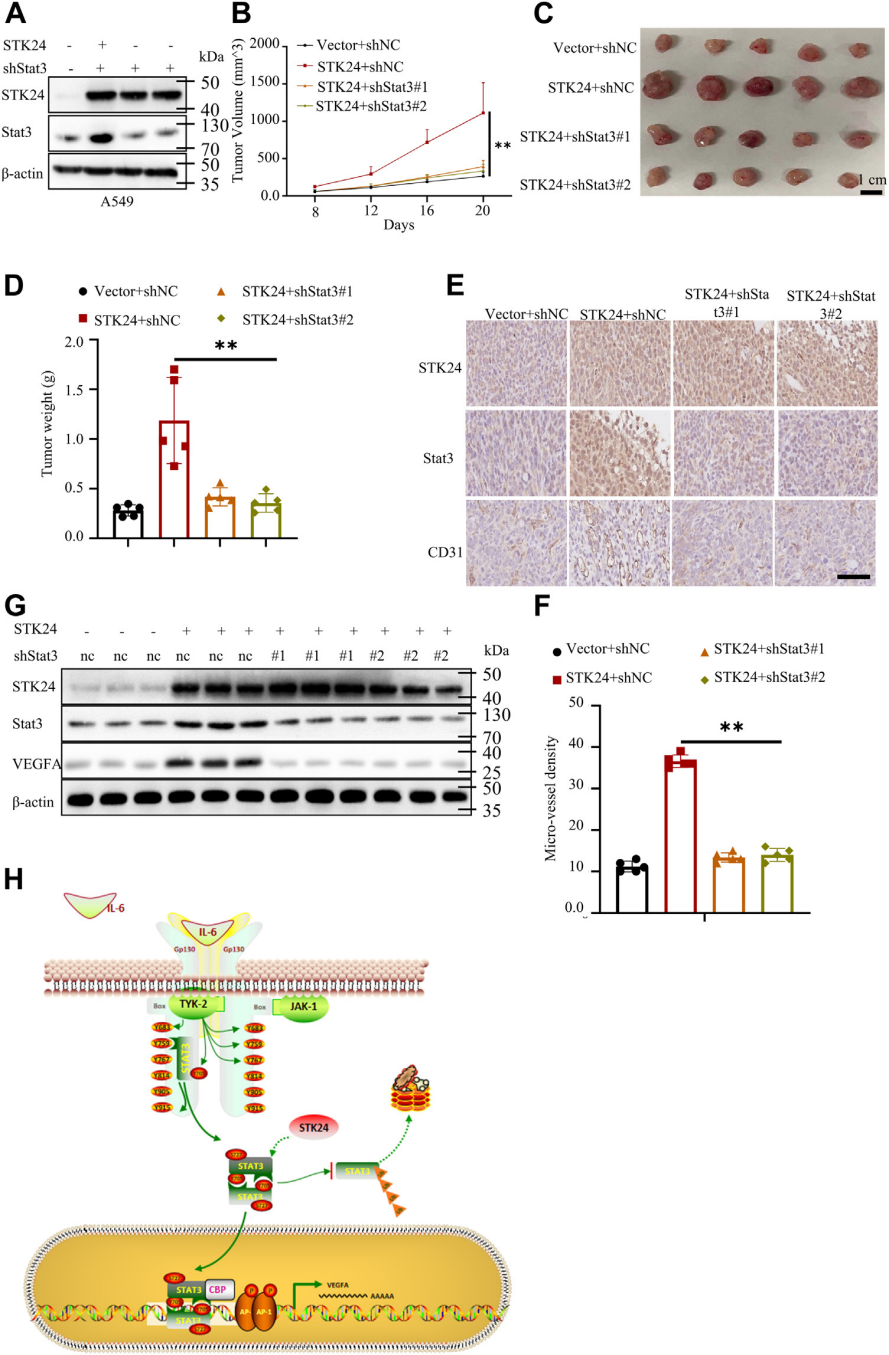
**STK24-mediated regulation of tumor angiogenesis is dependent on STAT3 *in vivo*.***A*, immunoblot analysis of indicated protein expression levels in A549 cells stably expressing vector+shnc, STK24+shnc, STK24+shSTAT3#1, or STK24+shSTAT3#2. *B*, shown tumor growth curve in *A* (n = 5); *p* = 0.0079 (STK24+shnc *versus* STK24+shSTAT3#1), 0.0079 (STK24+shnc *versus* STK24+shSTAT3#2), by Mann–Whitney test. *C*, shown representative tumor image in *A* (n = 5). *D*, statistical analysis of tumor weight in *A* (n = 5); *p* = 0.0079 (STK24+shnc *versus* STK24+shSTAT3#1), 0.0079 (STK24+shnc *versus* STK24+shSTAT3#2), by Mann–Whitney test. *E*, representative images for IHC analysis of tumors induced by indicated NSCLC cells in *A*; scale bar =100 μm. *F*, statistical analysis of microvessels density of indicated tumors in *A* (n = 5); *p* < 0.0001 (STK24+shnc *versus* STK24+shSTAT3#1 or STK24+shSTAT3#2), by Student’s *t* test. *G*, immunoblot analysis of protein expression levels of indicated tumors in *A*. *H*, the mechanism through which STK24 regulated the STAT3/VEGFA signaling pathway. All immunoblots were conducted three times, and consistent results were found. IHC, immunohistochemistry; NSCLC, nonsmall cell lung cancer; STK24, serine/threonine-protein kinase 24.

Mouse xenograft transplantation assays was conducted to validate the obtained findings on the relationship between STK24 and STAT3/VEGFA signaling pathway in tumor angiogenesis. Cancer cells stably expressing vector+shnc, STK24+shnc, STK24+shSTAT3#1, or STK24+shSTAT3#2 were subcutaneously injected into 4-week-old male nude BALB/c mice (Fig. 7*A*). Tumor volume was measured every 4 days, and after 20 days, all mice were sacrificed, tumors isolated and weighed. It was evident that tumors induced by cancer cells stably expressing STK24+shnc grew faster than tumors induced by cancer cells stably expressing vector+shnc. This effect was abolished by knocking down STAT3 expression (Fig. 7*B*). Similarly, tumors induced by cancer cells stably expressing STK24+shnc were much bigger than tumors induced by cancer cells stably expressing vector+shnc, with the loss of STAT3 reversing STK24-mediated tumor progression (Fig. 7, *C* and *D*). Results of IHC assays used to determine the density of microvessels in tumors showed that silencing of STAT3 abrogated STK24-mediated tumor angiogenesis (Fig. 7, *E* and *F*). Immunoblot analysis of protein expression level in tumors showed that tumors induced by cancer cells stably expressing STK24+shnc had higher STAT3 and VEGFA expression levels as compared with to tumors induced by cancer cells stably expressing vector+shnc. However, it was evident that silencing of STAT3 inhibited STK24-mediated VEGFA upregulation (Fig. 7*G*). In conclusion, the findings of this study demonstrated that STK24-mediated tumorigenesis, and tumor angiogenesis was dependent on STAT3/VEGFA signaling pathway.

## Discussion

Angiogenesis is a critical event for tumor progression and metastasis. In the current study, the mechanism of STK24-mediated regulation of angiogenesis in NSCLC was elucidated for the first time. It was found that STK24 activated STAT3 signaling pathway by inhibiting polyubiquitin–proteasomal–mediated degradation of STAT3. On the other hand, activation of STAT3 signaling pathway promoted tumor progression and angiogenesis through the upregulation of VEGFA expression.

High expression of STK24 is correlated with more aggressive breast cancer subtypes and poor prognosis (12). The STK24 regulates cell cycle by phosphorylating nuclear BDF2-related kinase two kinase at T442 (25). In addition, STK24 regulates cell migration through tyrosine phosphatase PTP-PEST and the adhesion complex molecule paxillin (14, 16, 18, 26). Further, STK24 enhances proliferation and tumorigenicity in a kinase-independent manner by regulating the VAV2/Rac1 signaling pathway (15). However, the expression of STK24 has been shown to be lower in gastric cancer as compared with matched normal tissues whereas loss of STK24 was found to promote tumorigenicity and distant metastasis in gastric cancer (26, 27). Nevertheless, the biological function of STK24 in lung cancer progress is still unclear. Besides, the role of STK24 in tumor angiogenesis is not explored in any tumors. In the present study, it was demonstrated that STK24 was upregulated in NSCLC tissues as compared with normal lung tissues, and NSCLC cells with higher expression of STK24 had stronger proliferation, migration, and invasion potential, which was on contrary to the function of STK24 in gastric cancer. These contradictory results may be attributed to the different experimental conditions and the different cancer subtypes.

STAT3 dysfunction appears to promote the proliferation, migration, and invasion of malignant tumors (28). Increased IL-6/JAK/STAT3 signaling has been observed in different types of tumors and tumor infiltrating immune cells, as well as that this has been associated with lack of response to immunotherapy (29). The IL-6/STAT3 signaling pathway upregulates factors involved in cancer cell proliferation (MYC, cyclin D1), angiogenesis (VEGFA, PDGF), and apoptosis (Bcl-XL) (30). Therefore, a deeper understanding of the IL-6/STAT3 signaling pathway is needed to promote the overall survival of patients with cancer. The IL-6/STAT3/VEGFA signaling pathway is activated in different cancers (31, 32, 33). Several pathways regulate the IL-6/STAT3/VEGFA signaling pathway, for instance, PARK2 inhibits tumor proliferation and angiogenesis by decreasing IL-6/STAT3/VEGFA signaling; Splice variant ΔNp63 activates the signaling pathway to upregulate hypoxia-inducible factor 1α, the secretion of VEGF and angiogenesis; IL-8 and IL-35 induce STAT3 phosphorylation; PTPN dephosphorylates STAT3 and inhibits STAT3 activation; MAGEC2/PDLIM2/Fbw7 interacts with STAT3 and inhibits its polyubiquitination and proteasomal degradation; TRAF6 negatively regulates the STAT3 signaling pathway by binding to STAT3 and mediating its ubiquitination (32, 34, 35, 36). In the current study, it was notably revealed the underlying function of SKT24 in tumor angiogenesis. Further, it was found that STK24 can positively regulate tumor angiogenesis. Furthermore, a novel regulation mechanism for STAT3, whereby STK24 interacts with STAT3 to prevent polyubiquitin–proteasomal–mediated degradation of STAT3, was also demonstrated. *In vitro* xenograft tumor assays confirmed that STK24 can positively administer STAT3/VEGFA signaling pathway. Furthermore, it was illustrated that STK24 mediated tumor proliferation and angiogenesis in a STAT3-dependent manner using *in vivo* and *in vitro* experiments. Remarkably, it was found that although STAT3 has been reported to regulate the levels of HIF1α expression, STK24 did not regulate levels of HIF1α expression (36). This discrepancy could be attributed to different experimental conditions and tumor models.

In conclusion, findings of the current study showed that STK24 acts as an oncogene, and the expression of STK24 in cancer cells is positively correlated with the proliferation, migration, invasion, and tumor angiogenesis potential of cancer cells. For mechanism of STK24 action, it was evident that STK24 prevents STAT3 from polyubiquitin–proteasomal–mediated degradation and STK24 regulated tumor angiogenesis through STAT3/VEGFA signaling pathway. The findings hence provide a novel insights into the mechanism of STK24 action and reveal the underlying mechanism of tumor angiogenesis, as well as provide the expected guidance to the future development of therapeutic strategies for cancer treatment.

## Experimental procedures

### Cell lines and antibodies

Three cell lines (H226, A549, and HUVECs) were obtained from the American Type Culture Collection (ATCC). Cell lines A549 and HUVECs were maintained with F-12K medium (21127022; Thermo Fisher Scientific), whereas H226 cells were cultured with 1640 medium (11875168; Thermo Fisher Scientific). Ten percent (v/v) of fetal bovine serum (12483012; Thermo Fisher SCIENTIFIC) was added into all media. As for F12K medium (500 ml), 50 mg stock heparin (H3393; Sigma) for a final concentration of 0.1 mg/ml was added, and 15 mg endothelial cell growth supplementary was also added. All cells were cultured in 95% air, 5% CO2 at 37 °C. Cells lines used in this study had been authenticated by cell line sequence report on December, 2020, December, 2021, and December, 2022. Cells used in this study were confirmed to be free from *mycoplasma*. All cells were cultured at good status. The growth rate of cells was fast, the cells were homogeneous and transparent, the intracellular particles were few, the vacuoles were not visible, the cell edges were clear, the suspended cells and fragments were not visible in the medium, and the culture medium was clear and transparent. All cells were cultured in 5% (v/v) CO2 at 37 °C. Antibodies for STK24 (3723; Cell Signaling Technology), B-actin (3700; Cell Signaling Technology), VEGFA (65373; Cell Signaling Technology), STAT3 (9239; Cell Signaling Technology), HIF1A (36169; Cell Signaling Technology), HIF1B (5537; Cell Signaling Technology), p-AKT^308^ (13038; Cell Signaling Technology), p-AKT^473^ (4060; Cell Signaling Technology), AKT (ab8805; Abcam), and CD31 (ab28364; Abcam), ubiquitin (ab134953; Abcam) were all commercially procured for the present study. The specificity of the antibody was validated by knockout testing.

### Real-time PCR

Total mRNAs were isolated from the indicated cancer cells using TRIzol (Invitrogen). The cDNAs were synthesized using ABScript II Reverse Transcriptase kit (RK21400; ABclonal). Quantitively real-time PCR was carried out using Genious 2X SYBR Green Fast qPCR Mix (RK21204; ABclonal). The primers used in the current study were: STAT3 forward: 5-CAGCAGCTTGACACACGGTA-3; STAT3 reverse: 5-AAACACCAAAGTGGCATGTGA-3; STK24 forward: 5-AGGCATTGACAATCGGACTCA-3; and STK24 reverse: 5- CTGACTCAGCACTGTGATTTCT-3.

### Cell lines construction

For STK24 knock-out cell lines construction, the three SgRNAs (bought from Shanghai Genechem Co, Ltd) (#1: 5-GGTCCATTGAAGAGCTGCGA-3; #2: 5-GCCACTCTACCTCATCCTGG-3; #3: 5-GAGAAGAGCCAGGCGTGCGG -3;) were cloned into pSpCas9(BB)-2A-Puro (PX459) V2.0 plasmids (62988; Addgene). Then, 2 μg sgRNAs-plasmids were transfected into A549 or H226 cell lines. After 48 h, 2 μg/ml puromycin (HY-B1743A; MedChemExpress) was applied to screen the infected cells until all the control cells died. 96-plate-wells were used for monoclonal screening. For Stat3 knock-down cell lines construction, the three shRNAs-Stat3 (bought from Shanghai Genechem Co, Ltd) (#1: F: 5- CCGG GCAGGGTTTGTCATTAATAATCTCGAGATTATTAATGACAAACCCTGC-3; R: 5-AAAAAGCAGGGTTTGTCATTAATAATCTCGAGATTATTAATGACAAACCCTGC-3 #2: F: 5- CCGGGACAGGTACAAGAGATGGAAGCTCGAGCTTCCATCTCTTGTACCTGTC-3; R: 5-AAAAAGACAGGTACAAGAGATGGAAGCTCGAGCTTCCATCTCTTGTACCTGTC-3; #3: F: 5- CCGGGTGGACAGAAATAAGATGAAAGCTCGAGCTTTCATCTTATTTCTGTCCA-3; R: 5-AAAAATGGACAGAAATAAGATGAAAGCTCGAGCTTTCATCTTATTTCTGTCCAC-3) were cloned into pLKO.1 plasmids (8453; Addgene). Then pLKO.1-shRNAs, MD2-G, and PPAX three packing system was applied to generate lentivirus in HEK293 T cells. The lentivirus subsequently was added into A549 or H226 cell lines. After 48 h, 2 μg/ml puromycin was applied to screen the infected cells until all the control cells died. For STK24 overexpression cell lines construction, a cDNA coding STK24 was cloned into pLVX-Vector plasmids (bought from Shanghai Genechem Co,Ltd). Then pLVX-STK24, MD2-G, and PPAX three packing system was applied to generate lentivirus in HEK293T cells. The lentivirus subsequently was added into A549 or H226 cell lines. After 48 h, 2 μg/ml puromycin was applied to screen the infected cells until all the control cells died. Mut Express MultiS Fast Mutagenesis Kit V2 (C215-01; Vazyme) was used for pLVX-STK24 K53R plasmid construction. The successful construction of identified cells was confirmed by RT-PCR or Western blot assays.

### Western blot and immunoprecipitation

Cultured cells were collected and washed using cold PBS (C0221A; Beyotime). The cells were then lysed using NP-40 lysis buffer (P0013F; Beyotime). Protein concentration was measured using bicinchoninic acid assay kit (23225; Thermo). The proteins were separated through electrophoresis in premade sodium dodecyl sulfate-polyacrylamide minigels and were then transferred to PVDF membranes (88520; Thermo). The membranes were incubated overnight with primary antibodies (dilution 1:1000) at 4 °C before incubation with the secondary antibodies (dilution 1:1000). Protein bands were detected using chemiluminescence (GelView 6000 Pro; BLT). For immunoprecipitation, primary antibodies were incubated with Protein L Magnetic Beads (HY-K0205; MCE) at room temperature for 2 h. The whole cell lysates were then incubated overnight with beads at 4 °C, followed by Western blot analysis. For denaturing immunoprecipitation, cells were lysed in 1% SDS lysis buffer and boiled for 20 min, and the lysates were centrifuged and diluted 1:10 with lysis buffer. The diluted lysates were followed by immunoprecipitation.

### GST pull-down assay

GST-STK24 fusion protein was purified from HEK293T cells using Anti-GST Magnetic Beads (HY-K0222; MedChemExpress), and His-STAT3 fusion protein was isolated from BL21 using Ni bead (C650033; Sangon), purified recombinant His-STAT3 protein was co-incubated with purified GST or recombinant GST-STK24 protein over night at 4 °C, followed by immunoprecipitation using Anti-GST Magnetic Beads.

### Immunohistochemistry

This study was approved by the Huazhong University of Science and Technology Ethics Committee. The formed consents had been obtained from NSCLC patients. We strictly obeyed Helsinki principles in this study. Formalin-fixed paraffin-embedded tissues were cut into 4 mm section. Primary antibodies (dilution 1:100) were applied for IHC assays. Two experienced pathologists independently evaluated the obtained immunostaining results. IHC staining was quantified by multiplying the proportion score with the intensity score. Whereby the proportion score reflected the fraction of positively stained tumor cells: 1 (<10%); 2 (10–50%); 3 (50–75%); 4 (>75%), whereas the intensity score indicated the staining intensity (0, no staining; 1, weak; 2, intermediate and 3, strong). The obtained staining score ranged between 0 and 12.

### CCK8 assays

A total of between 1000 and 3000 pretreated cells were seeded in 96-well plates. HUVECs were treated with CM. The CCK8 assays were carried out using Cell Counting Kit-8 (RM02823; ABclonal) using automatic microplate reader (synergy 2; BioTek) according to instructions provided by the manufacturer.

### Cell cycle analysis

The cells were washed with cold PBS and then fixed overnight with 80% ethanol at −20 °C. Thereafter, the cells were washed with cold PBS and stained with PI (40710ES03; YEASEN) for 15 min at room temperature. The distribution analysis of the cell cycle was carried out using the Becton-Dickinson FACScan System.

### Transwell assays

The transwell chambers (CLS3450-24EA) were purchased from Merck company. The cells were seeded in serum-free medium at a density of between 1 and 4∗10ˆ4 per well into the upper chamber with or without Matrigel (354234; CORNING). A 500 μl conditional medium was added into the lower chamber. Further, Mitomycin C (10 μg/ml; M5353; Merck) was used to inhibit cell proliferation. After 10 to 20 h, the cells were washed thrice with PBS, fixed with 4 % formaldehyde (1004965000; Merck) for 30 min, and then stained with 0.1% crystal violet (C6158; Merck). Cell images were obtained using a microscope (BX53; OLYMPUS).

### Wound healing assays

A linear wound was made using a 200-μl sterile plastic pipette tip after cells reached 95 % confluency. Mitomycin C (10 μg/ml) was used to inhibit cell proliferation. After 12 h (Fig. 2) or 24 h (Figs. S3 and S5), cells were washed twice using PBS, and then the wound sizes were observed as well as measured at the indicated times (BX53; OLYMPUS).

### Enzyme-linked immunosorbent assay

ELISA assays were carried out using ELISA kits (RK00023; ABclonal) using automatic microplate reader (synergy 2; BioTek) and according to the instructions provided by the manufacturer.

### Tube formation assay

Matrigel (100 μl) (354234; CORNING) was added into the 96-well plates. 1 to 4∗10^ˆ^4 HUVECs were seeded into each well and cultured with conditional mediums for between 10 and 16 h. Images were captured using a microscope.

### Mouse xenograft experiments

The mouse xenograft assays were approved by the Animal Care and Use Committee of Tongji Hospital. Four-week-old male BALB/c mice were purchased from Beijing Huafukang Bioscience Company. Husbandry of mice obeyed the rules of Breeding and management of SPF mice. We designed the mouse experiments according to the triple blind method. Before mouse experiments, mice were grouped randomly. A total of 1 to 2 × 10^6^ indicated cells were subcutaneously injected into the back of each 5- to 6-week-old male BALB/c. Each mouse was injected one tumor at the same place. Tumor volume was also measured at the indicated time using formulae: 0.5 × Length (L) × Width (W)^2^. After indicated time, all mice were sacrificed, and all tumor xenografts were isolated and weighed. Then, all xenografts were cut in half. One half of tumor was fixed in 4 % formaldehyde for IHC assays, and the other was preserved in −80 °C for further assays.

### Database

Databases for The Cancer Genome Atlas (TCGA; http://xena.ucsc.edu/welcome-to-ucsc-xena/) and Human Protein Atlas database (https://www.proteinatlas.org) were used for statistical analysis in the present study.

### Statistical analysis

All the data obtained in the present study were analyzed using SPSS 20.0 software and specificity. Data were shown as mean ± SD. Kaplan–Meier curve was tested by Log-rank (Mantel–Cox) test. Correlation analysis was calculated by Pearson's r test. Differences between two groups were compared using the Student’s *t* test or Mann–Whitney test, while the differences among multiple groups were compared using ANOVA. *p* value <0.05 was considered statistically significant.

## Data availability

Data are available within the article or its supplementary materials.

## Supporting information

This article contains supporting information.

## Conflict of interest

The authors declare that they have no conflict of interest with the contents of the article.

## References

[1] Duma N., Santana-Davila R., Molina J.R. (2019). Non-small cell lung cancer: epidemiology, screening, diagnosis, and treatment. Mayo Clin. Proc..

[2] Gridelli C., Rossi A., Carbone D.P., Guarize J., Karachaliou N., Mok T. (2015). Non-small-cell lung cancer. Nat. Rev. Dis. Primers.

[3] Hirsch F.R., Scagliotti G.V., Mulshine J.L., Kwon R., Curran W.J., Wu Y.-L. (2017). Lung cancer: current therapies and new targeted treatments. Lancet.

[4] Li T., Kang G., Wang T., Huang H. (2018). Tumor angiogenesis and anti-angiogenic gene therapy for cancer. Oncol. Lett..

[5] Jing X., Yang F., Shao C., Wei K., Xie M., Shen H. (2019). Role of hypoxia in cancer therapy by regulating the tumor microenvironment. Mol. Cancer.

[6] Ciccarese C., Iacovelli R., Porta C., Procopio G., Bria E., Astore S. (2021). Efficacy of VEGFR-TKIs plus immune checkpoint inhibitors in metastatic renal cell carcinoma patients with favorable IMDC prognosis. Cancer Treat. Rev..

[7] Eswarappa S.M., Fox P.L. (2015). Antiangiogenic VEGF-Ax: a new participant in tumor angiogenesis. Cancer Res..

[8] Detmar M. (2000). Tumor angiogenesis. J. Investig. Dermatol. Symp. Proc..

[9] Goel H.L., Mercurio A.M. (2013). VEGF targets the tumour cell. Nat. Rev. Cancer.

[10] Tabernero J., Macarulla T., Ramos F.J., Baselga J. (2005). Novel targeted therapies in the treatment of gastric and esophageal cancer. Ann. Oncol..

[11] Serrano J.J., Delgado B., Medina M.Á. (2020). Control of tumor angiogenesis and metastasis through modulation of cell redox state. Biochim. Biophys. Acta Rev. Cancer.

[12] Thompson B.J., Sahai E. (2015). MST kinases in development and disease. J. Cell Biol..

[13] Iglesias C., Floridia E., Sartages M., Porteiro B., Fraile M., Guerrero A. (2017). The MST3/STK24 kinase mediates impaired fasting blood glucose after a high-fat diet. Diabetologia.

[14] Yang J., Jiang Q., Yu X., Xu T., Wang Y., Deng J. (2020). STK24 modulates excitatory synaptic transmission in epileptic hippocampal neurons. CNS Neurosci. Ther..

[15] Cho C.-Y., Lee K.-T., Chen W.-C., Chang S., Huang H.-L., Hsu H.-P. (2016). MST3 promotes proliferation and tumorigenicity through the VAV2 Rac1 signal axis in breast cancer. Oncotarget.

[16] Huang N., Lin W., Shi X., Tao T. (2018). STK24 expression is modulated by DNA copy numbermethylation in lung adenocarcinoma and predicts poor survival. Future Oncol..

[17] Wang R., Wu S.T., Yang X., Qian Y., Choi J.P., Gao R. (2021). Pdcd10-Stk24/25 complex controls kidney water reabsorption by regulating Aqp2 membrane targeting. JCI Insight.

[18] Qin Q., Shou J., Li M., Gu M., Meng Z., Xu P. (2021). Stk24 protects against obesity-associated metabolic disorders by disrupting the NLRP3 inflammasome. Cell Rep..

[19] Ultanir S.K., Yadav S., Hertz N.T., Oses-Prieto J.A., Claxton S., Burlingame A.L. (2014). MST3 kinase phosphorylates TAO1/2 to enable Myosin Va function in promoting spine synapse development. Neuron.

[20] Huang C.Y., Wu Y.M., Hsu C.Y., Lee W.S., Lai M.D., Lu T.J. (2002). Caspase activation of mammalian sterile 20-like kinase 3 (Mst3). Nuclear translocation and induction of apoptosis. J. Biol. Chem..

[21] Liu T., Wu H.J., Liang Y., Liang X.J., Huang H.C., Zhao Y.Z. (2016). Tumor-specific expression of shVEGF and suicide gene as a novel strategy for esophageal cancer therapy. World J. Gastroenterol..

[22] Fleming M.R., Xiao L., Jackson K.D., Beckman J.A., Barac A., Moslehi J.J. (2021). Vascular impact of cancer therapies: the case of BTK (Bruton tyrosine kinase) inhibitors. Circ. Res..

[23] Shu Y., Cheng P. (2020). Targeting tumor-associated macrophages for cancer immunotherapy. Biochim. Biophys. Acta Rev. Cancer.

[24] De Palma M., Biziato D., Petrova T.V. (2017). Microenvironmental regulation of tumour angiogenesis. Nat. Rev. Cancer.

[25] Stegert M.R., Hergovich A., Tamaskovic R., Bichsel S.J., Hemmings B.A. (2005). Regulation of NDR protein kinase by hydrophobic motif phosphorylation mediated by the mammalian Ste20-like kinase MST3. Mol. Cell. Biol..

[26] Chen Y.-L., Wang C.-Y., Fang J.-H., Hsu H.-P. (2021). Serinethreonine-protein kinase 24 is an inhibitor of gastric cancer metastasis through suppressing CDH1 gene and enhancing stemness. Am. J. Cancer Res..

[27] Hsu H.P., Wang C.Y., Hsieh P.Y., Fang J.H., Chen Y.L. (2020). Knockdown of serine/threonine-protein kinase 24 promotes tumorigenesis and myeloid-derived suppressor cell expansion in an orthotopic immunocompetent gastric cancer animal model. J. Cancer.

[28] Chai E.Z., Shanmugam M.K., Arfuso F., Dharmarajan A., Wang C., Kumar A.P. (2016). Targeting transcription factor STAT3 for cancer prevention and therapy. Pharmacol. Ther..

[29] Yu H., Lee H., Herrmann A., Buettner R., Jove R. (2014). Revisiting STAT3 signalling in cancer: new and unexpected biological functions. Nat. Rev. Cancer.

[30] Laudisi F., Cherubini F., Monteleone G., Stolfi C. (2018). STAT3 Interactors as potential therapeutic targets for cancer treatment. Int. J. Mol. Sci..

[31] Liu Y., Liao S., Bennett S., Tang H., Song D., Wood D. (2021). STAT3 and its targeting inhibitors in osteosarcoma. Cell Prolif..

[32] Zhao J., Du P., Cui P., Qin Y., Hu C., Wu J. (2018). LncRNA PVT1 promotes angiogenesis via activating the STAT3/VEGFA axis in gastric cancer. Oncogene.

[33] Johnson D.E., O'Keefe R.A., Grandis J.R. (2018). Targeting the IL-6/JAK/STAT3 signalling axis in cancer. Nat. Rev. Clin. Oncol..

[34] Ma J.H., Qin L., Li X. (2020). Role of STAT3 signaling pathway in breast cancer. Cell Commun. Signal..

[35] Nie X.H., Ou-yang J., Xing Y., Li D.Y., Dong X.Y., Liu R.E. (2015). Paeoniflorin inhibits human glioma cells via STAT3 degradation by the ubiquitin-proteasome pathway. Drug Des. Devel. Ther..

[36] Zhao T., Jin F., Xiao D., Wang H., Huang C., Wang X. (2020). IL-37/STAT3/HIF-1alpha negative feedback signaling drives gemcitabine resistance in pancreatic cancer. Theranostics.

